# Association of Genetic Polymorphisms with Gestational Diabetes in a Kazakh Population: A Case–Control Study

**DOI:** 10.3390/diagnostics16050663

**Published:** 2026-02-25

**Authors:** Laura Danyarova, Gulnara Svyatova, Galina Berezina, Rustem Tuleutayev, Balnur Sultanova, Assel Taigulova, Aigul Sartayeva, Moldir Zhekeyeva, Indira Karibayeva

**Affiliations:** 1 National Center of Endocrinology and Diabetes, JSC Research Institute of Cardiology and Internal Diseases, Almaty 050012, Kazakhstan; la.danyarova@gmail.com (L.D.); moldirzhekeeva@gmail.com (M.Z.); 2 Scientific Department of the Center for Molecular Medicine, Center for Molecular Medicine, Almaty 050026, Kazakhstan; gsvyatova1@mail.ru; 3 Department of Strategy and Science, JSC Scientific Center of Obstetrics, Gynecology and Perinatology, Almaty 050020, Kazakhstan; gberezina54@mail.ru; 4 JSC Research Institute of Cardiology and Internal Diseases, Almaty 050000, Kazakhstan; rustemtuleutayev@gmail.com; 5 Department of Internal Medicine, Division of Graduate and Continuing Education, JSC Research Institute of Cardiology and Internal Diseases, Almaty 050000, Kazakhstan; 6 Department of Pediatrics, Semey Medical University, Semey 071400, Kazakhstan; taigulovaassel@gmail.com; 7 Department of General Medical Practice No. 2, West Kazakhstan Marat Ospanov Medical University, Aktobe 030000, Kazakhstan; sartaeva.aigul1@gmail.com; 8 Department of Health Policy and Community Health, Jiann-Ping Hsu College of Public Health, Georgia Southern University, Statesboro, GA 30460, USA

**Keywords:** gestational diabetes mellitus, genetic polymorphisms, *IGF2BP2*, *CDKAL1*, single-nucleotide polymorphisms, gene–gene interaction, Kazakh population, case–control study, glucose metabolism

## Abstract

**Background**: Gestational diabetes mellitus (GDM) poses a growing public health challenge worldwide due to its increasing prevalence, associated pregnancy complications, and long-term metabolic risks for both mothers and offspring. Genetic factors are known to contribute to GDM susceptibility, yet little is known about their relevance in ethnic Kazakh population. The primary objective of this study was to evaluate associations between selected candidate SNPs involved in β-cell function and the risk of GDM in a Kazakh cohort. Secondary objectives included the assessment of potential gene–gene interactions. **Methods**: We conducted a case–control study among 365 pregnant Kazakh women. Of these, 217 were diagnosed with GDM, and 148 had normal glucose tolerance. Clinical and genealogical data were collected. Eight candidate SNPs that were previously associated with GDM or glucose metabolism were genotyped. Logistic regression was used to assess associations between SNPs and GDM risk. Gene–gene interactions were evaluated using multifactor dimensionality reduction (MDR). **Results**: In univariate analysis, *MTNR1B* rs10830963 demonstrated a statistically significant association under the additive model (OR 0.61, 95% CI 0.42–0.89), indicating a potential protective effect of the C allele. However, this association was not statistically significant after multivariable adjustment (adjusted OR 0.58, 95% CI 0.32–1.03) and correction for multiple testing. In the adjusted analysis, *TCF7L2* rs7903146 showed a significant association under the dominant model (adjusted OR 2.29, 95% CI 1.01–5.46); however, this finding did not remain significant following FDR correction. MDR analysis showed that the best two-locus model included *IGF2BP2* rs4402960 and *CDKAL1* rs7754840 (CVC = 6/10; testing accuracy = 0.558; permutation *p* < 0.001). The most stable interaction was observed for the three-locus model comprising *IGF2BP2* rs4402960, *MTNR1B* rs10830963, and *PPARG* rs1801282 (CVC = 9/10; testing accuracy = 0.576; permutation *p* < 0.001). **Conclusions**: The findings suggest that common variants in *IGF2BP2*, *CDKAL1*, *MTNR1B*, *TCF7L2*, *PPARG,* and *GCK* do not exert strong individual effects on GDM susceptibility in this cohort of ethnic Kazakh women. Instead, the results are more consistent with a modest polygenic architecture characterized by small effect sizes and possible weak gene–gene interactions. MDR analysis identified statistically significant interaction models; however, their limited predictive accuracy indicates that these findings should be interpreted as exploratory.

## 1. Introduction

Gestational diabetes mellitus (GDM), defined as glucose intolerance first identified during pregnancy, represents an escalating global public health concern [1]. According to the International Diabetes Federation, in 2024, approximately one in six live births worldwide was affected by GDM [2], with substantial regional variation driven by differences in ethnicity, maternal age, body mass index (BMI), and diagnostic criteria. GDM is associated with a wide range of adverse maternal and neonatal outcomes, including preeclampsia, fetal macrosomia, neonatal hypoglycemia, preterm birth, cesarean delivery, and increased risk of neonatal intensive care unit (NICU) admission [3,4,5]. Beyond pregnancy, women with GDM face a markedly increased risk of type 2 diabetes mellitus (T2DM) [6,7], while offspring exposed to maternal hyperglycemia are predisposed to obesity, impaired glucose tolerance, and cardiovascular disease later in life [8].

The economic burden of GDM is substantial due to increased healthcare utilization, intensive prenatal monitoring, and long-term disease consequences for both mother and child. Evidence from large-scale economic evaluations highlights the financial implications of both early detection and delayed management. The TOBOGM trial, a multicenter randomized controlled study conducted between 2017 and 2022 across 17 hospitals in Australia, Austria, India, and Sweden, demonstrated that early diagnosis and treatment of GDM not only improved clinical outcomes but also showed a tendency toward cost savings compared with standard care, with an estimated mean cost difference of −$1373 (95% CI: −$3749 to $642) [9]. Similarly, population-level data from China indicate a substantial societal impact of GDM, with the annual economic burden estimated at approximately ¥19.36 billion (US $5.59 billion) in 2015 [10]. In addition to direct and indirect costs, GDM was associated with considerable health losses, corresponding to an estimated 260,000 quality-adjusted life years (QALYs) lost annually [10]. Together, these findings underscore the significant economic and public health consequences of GDM and highlight the importance of effective prevention, early detection, and targeted intervention strategies.

Preventive strategies for GDM have predominantly targeted modifiable risk factors, including lifestyle and metabolic interventions implemented before or during pregnancy. Identifying risk factors that can be modified is essential for lowering the likelihood of developing GDM and minimizing related health complications [11]. Evidence from systematic reviews indicates that interventions based on dietary modification, increased physical activity, and combined diet–exercise programs, as well as pharmacological and nutritional approaches such as metformin and myoinositol supplementation, are associated with a reduced incidence of GDM compared with standard care [12,13]. However, despite these preventive efforts, the broader burden of dysglycemia will continue to rise. Prediction models show that by 2050, in women, the global incidence, disability-adjusted life years (DALYs), and mortality associated with type 2 diabetes (T2D) will all increase over time—particularly among younger age groups—despite declining mortality trends in higher Socio-demographic Index (SDI) countries [14].

Genetic susceptibility contributes to interindividual variability in pancreatic β-cell compensation during pregnancy. Genome-wide association studies (GWASs) indicate that many loci associated with GDM overlap with those implicated in type 2 diabetes, particularly genes regulating insulin secretion rather than peripheral insulin sensitivity. This suggests that genetically determined β-cell vulnerability plays a central role in GDM pathogenesis. Single-nucleotide polymorphisms (SNPs) in genes such as *IGF2BP2*, *CDKAL1*, *MTNR1B*, *TCF7L2*, and *PPARG* have been consistently associated with GDM and T2DM in diverse populations [15,16]. For example, *TCF7L2* rs7903146 has been linked to increased GDM risk in European cohorts [17], and *MTNR1B* rs10830963 has been associated with fasting glucose and GDM in Chinese populations [18]. Similarly, *CDKAL1* rs7754840 and *PPARG* rs1801282 variants have been implicated in GDM susceptibility in multiple ethnic groups [19,20]. However, no significant association between *IGF2BP2* rs4402960 and GDM was observed in a recent meta-analysis [21].

Research on the genetic epidemiology of GDM in Central Asia, particularly in Kazakhstan, remains limited, despite the rising burden of both DM and GDM in the country. Kazakhstan represents a historically admixed population shaped by Turkic, Mongolian, Slavic, and Central Asian ancestries [22]. Such genetic heterogeneity may influence allele frequencies, linkage disequilibrium patterns, and gene–environment interactions relevant to metabolic disease. Although numerous single-nucleotide polymorphisms (SNPs) have been associated with GDM and type 2 diabetes (T2D) in global studies, there is limited evidence regarding their relevance in the Kazakh population. Recent studies have begun to address this gap. For example, a 2024 case–control study by Sikhayeva et al. examined ADIPOQ gene polymorphisms and found significant associations with T2D and obesity among Kazakh individuals, suggesting a genetic basis for metabolic risk in this population [23]. Additional investigations into *PPARG*, *TCF7L2*, and *ANRIL* gene variants have revealed associations with prediabetes and coronary artery disease among Kazakh patients with diabetes, underscoring the importance of population-specific genetic research [24].

Notably, a 2022 study by Svyatova et al. examined allele frequencies of eight SNPs across seven genes related to glucose metabolism and insulin signaling in healthy Kazakh individuals [25]. The study reported high frequencies of risk alleles in *MTNR1B* (rs10830963), *TCF7L2* (rs7903146)*,* and *GCK* (rs4607517), suggesting potential genetic susceptibility to GDM in this population. A subsequent genome-wide study analyzed *TCF7L2* (rs7903146) and *PPARG* (rs1801282) variants in 1800 ethnically Kazakh individuals and found that the protective G allele of *PPARG* occurred at a frequency of 13.8%, while the risk-associated T allele of *TCF7L2* was present in 15.2% of the sample, indicating relevance for prediabetes risk [26]. Both studies have described allele frequencies of metabolic risk variants in healthy Kazakh cohorts; however, detailed analyses examining genotype–phenotype associations in GDM are lacking.

We hypothesized that variants in genes regulating pancreatic β-cell function would be associated with altered risk of GDM in a Kazakh cohort and that population substructure might influence genotype distribution. The primary objective of this study was to evaluate associations between selected candidate SNPs involved in β-cell function and the risk of GDM in a Kazakh cohort. Secondary objectives included the assessment of potential gene–gene interactions.

## 2. Materials and Methods

### 2.1. Study Design and Study Population

This study employed a case–control design to investigate clinical, genealogical, and genetic factors associated with GDM. Participants were recruited from obstetric clinics across four regions in Kazakhstan—Aktobe, Almaty, Almaty Region, and Semey—from April 2022 to June 2023 (15 months). A total of 365 pregnant women were enrolled between 24 and 28 weeks of gestation. Among them, 217 women were diagnosed with GDM based on the World Health Organization (WHO) 2013 criteria using a 75 g oral glucose tolerance test (OGTT), and 148 women with normal glucose tolerance served as non-GDM controls. Written informed consent was obtained from all participants prior to enrollment. Pregnant women were recruited at 24–28 weeks’ gestation prior to the classification of GDM status. Diagnosis was established according to the WHO 2013 criteria based on a 75 g oral glucose tolerance test (OGTT). Participants were consecutively enrolled, and no matching was performed. No a priori SNP-specific power calculation was performed; therefore, the study should be considered exploratory.

Inclusion criteria for both groups included age between 18 and 45 years, Kazakh ethnicity, singleton pregnancy, and availability of complete clinical and biochemical data. Exclusion criteria included pre-existing diabetes mellitus, chronic kidney or liver disease, autoimmune disorders, or other significant metabolic conditions. All participants provided written informed consent prior to enrollment. The study was approved by the Institutional Ethics Committee of the Asfendiyarov Kazakh National Medical University (Protocol No. 5 (111) dated 28 April 2021) and conducted in accordance with the Declaration of Helsinki.

### 2.2. Genealogical Data

In the Kazakh context, such self-reported genealogical identity is considered highly reliable because genealogical knowledge (shezhire) is deeply embedded in cultural and social life. From childhood, Kazakhs are taught to recite their zheti ata (“seven ancestors”), which encompasses paternal lineage (ata), tribe (ru), and socio-territorial subdivision (zhuz) [27]. This tradition serves both as a safeguard against close-kin marriage and as a marker of social cohesion and ancestral geography.

Genealogical records in the form of shezhire exist in both public and private formats. Publicly accessible shezhire sources typically provide extensive historical lineage information beginning at the zhuz level and extending through ru and their descendants, whereas privately maintained shezhire records often contain more detailed and up-to-date information on recent generations [28].

Historian Asfendiarov argued that the formation of the Kazakh zhuzes reflected distinct economic, cultural, and historical conditions shaped by the natural division of Kazakhstan into the Semirechie, Western, and Central regions [29]. Kishi zhuz (Junior) is concentrated in Western Kazakhstan. Orta zhuz (Middle) is dominant in the Central regions. Uly zhuz (Senior) is historically centered in southern Kazakhstan (Semirechie) [30].

In addition to the three main zhuz, the genealogical information includes Tore and Kozha, two historically distinct lineages [31]. Tore genealogies are traditionally considered descendants of Genghis Khan, while those of Kozha trace their ancestry to Islamic religious scholars. These groups do not belong to the formal zhuz classification and were therefore recorded and analyzed separately. Importantly, zhuz affiliation in this study is interpreted as a sociocultural indicator of potential population substructure rather than a direct measure of genetic ancestry. Because genome-wide ancestry-informative markers were not assessed, residual population stratification cannot be excluded. Zhuz affiliation was not included as a covariate in primary logistic regression models due to limited sample size and multicollinearity with the region.

### 2.3. Genealogical Data Collection

Participants provided genealogical information via structured questionnaires at enrollment. They self-reported the zhuz (socio-territorial subdivision) affiliation of their maternal and paternal grandparents. In this study, the inclusion of participants from four geographically distinct regions, Almaty, Almaty Region (Semirechie), Semey (East and Central), and Aktobe (Western), was strategically designed to reflect the diversity of genealogical backgrounds and ensure a representative sampling of all major *zhuz* affiliations. Almaty city, as a metropolitan hub, is demographically diverse and includes representatives from all zhuz.

### 2.4. Clinical Measurements

Clinical assessments were conducted at the time of enrollment. Anthropometric measurements included height, weight, and body mass index (BMI), which was calculated as weight in kilograms divided by height in meters squared (kg/m^2^).

### 2.5. DNA Extraction and Genotyping

The eight candidate SNPs included in this study were selected based on prior genome-wide association signals in GDM or T2D, biological plausibility in β-cell or insulin signaling pathways, and reported minor allele frequencies sufficient to permit analysis in Central Asian populations. Preference was given to variants with replicated associations across multiple ethnic cohorts. A total of eight SNPs were selected for genotyping based on their previously reported associations with insulin signaling and glucose homeostasis. These included two variants in *Insulin-Like Growth Factor 2 mRNA-Binding Protein 2* (*IGF2BP2*; rs1470579, TaqMan assay ID: C___2165184_10; rs4402960, C___2165199_10), and one SNP each in *Cyclin-Dependent Kinase 5 Regulatory Subunit-Associated Protein 1-Like 1* (*CDKAL1*; rs7754840, C__29246232_10), *Insulin Receptor Substrate 1* (*IRS1*; rs1801278, C___2384392_20), *Melatonin Receptor 1B* (*MTNR1B*; rs10830963, C___3256858_10), *Transcription Factor 7-Like 2* (*TCF7L2*; rs7903146, C__29347861_10), *Peroxisome Proliferator-Activated Receptor Gamma* (*PPARG*; rs1801282, C___1129864_10), and *Glucokinase* (*GCK*; rs4607517, C__27917691_10).

Peripheral blood samples were collected from the cubital vein into EDTA-containing vacutainer tubes. Genomic DNA was extracted using magnetic M-PVA bead separation on the Prepito^®^ automated nucleic acid extraction platform (PerkinElmer, Wallac, Finland), with the Prepito^®^ DNA CytoPure reagent kit.

DNA purity was assessed by measuring absorbance ratios at 260/280 nm using a NanoDropLite UV spectrophotometer (Thermo Fisher Scientific, Waltham, MA, USA). Samples with absorbance ratios around 1.8 were considered pure. The DNA was suspended in nuclease-free water and stored at –40 °C until further analysis. Genotyping was performed within ten days of extraction using a real-time polymerase chain reaction (PCR) on the StepOnePlus™ system (Applied Biosystems, Foster City, CA, USA), with TaqMan^®^ allele discrimination assays (Thermo Fisher Scientific, Waltham, MA, USA). All assays were conducted according to the manufacturer’s protocol using standard TaqMan reagents and TE buffer (pH 8.0, 10 mM Tris-HCl, 1 mM EDTA; Invitrogen, Thermo Fisher Scientific, Waltham, MA, USA). The quality of DNA used for genotyping was confirmed by purity ratios ranging from 1.7 to 2.0. High-quality DNA samples demonstrated clear clustering on allele discrimination plots. Any outliers that fell outside defined clusters were excluded from analysis, and DNA extraction and PCR were repeated. As part of quality control, 5% of samples were randomly re-genotyped, confirming 100% concordance and ensuring genotyping reliability. Genotyping call rates exceeded 95% for all SNPs. Minor allele frequencies (MAFs) were calculated in controls. Hardy–Weinberg Equilibrium (HWE) was assessed in controls using the exact test. Variables deviating from HWE were excluded from further analysis.

### 2.6. Statistical Analysis Plan

All statistical analyses were conducted using R version 4.5.1 (13 June 2025) [32] within the RStudio environment (version 2025.9.0.387) [33]. Statistical significance was set at a two-sided *p*-value of less than 0.05 for all comparisons. Descriptive analyses were first performed to summarize the baseline characteristics of the study population. Continuous variables were assessed for normality using the Shapiro–Wilk test. Non-normally distributed variables were reported as medians with interquartile ranges (IQRs). Categorical variables were described using frequencies and percentages.

To compare baseline characteristics between participants with GDM and non-GDM controls, appropriate statistical tests were selected based on data type and distribution. For continuous variables, comparisons were made using the non-parametric Mann–Whitney U test. Categorical variables were analyzed using Pearson’s chi-square test or Fisher’s exact test, as appropriate.

To examine the association between selected single-nucleotide polymorphisms (SNPs) and the risk of GDM, both univariate and multivariate logistic regression analyses were conducted. Crude odds ratios (ORs) and adjusted odds ratios (AORs), along with 95% confidence intervals (CIs), were estimated. Multivariate models were adjusted for potential confounders, including maternal body mass index (BMI), education, region, employment status, and parity. To account for multiple testing across SNPs, false discovery rate (FDR) correction was applied using the Benjamini–Hochberg procedure.

To assess potential epistatic (gene–gene) interactions among the studied SNPs, multifactor dimensionality reduction (MDR) analysis was performed in R [34]. Ten-fold cross-validation was used to validate model stability, and the best models were selected based on cross-validation consistency (CVC), testing accuracy, and permutation-based *p*-values. Post hoc statistical power was estimated based on the observed sample size (217 cases and 148 controls), case–control ratio, and typical minor allele frequencies. Assuming a two-sided α of 0.05 under an additive genetic model, the study had approximately 80% power to detect odds ratios ≥ 1.6, whereas power was substantially lower for detecting modest effect sizes (OR ≤ 1.3). All data analyzed in the present study are provided in Supplementary Table S1.

## 3. Results

### Original Study Results

Table 1 summarizes the baseline characteristics of participants with GDM and non-GDM controls. The median age of GDM patients was 32.0 years (IQR: 27.0–35.0), compared to 30.0 years (IQR: 27.0–35.0) among controls, with no significant difference observed (*p* = 0.59). GDM patients had significantly higher BMI values than controls, with 29.49 [24.52, 34.08] and 25.34 [21.77, 31.2], respectively (*p = 0.0187*). Significant differences were found between groups in the distribution of genealogical *zhuz* affiliations for both maternal and paternal grandparents (all *p*-values < 0.001). A higher proportion of GDM patients belonged to the Kishi *zhuz*, whereas controls were more frequently associated with the Orta *zhuz*. Marital status did not differ significantly between groups, with the majority of both GDM patients (94%) and controls (97%) being married (*p* = 0.242). Educational attainment, however, showed a statistically significant difference (*p* = 0.038), with a higher proportion of controls having a bachelor’s degree or higher (66%) compared to GDM patients (54%). Regional distribution varied markedly (*p* < 0.001). Participants with GDM were more likely to reside in Aktobe (44%) or the Almaty Region (27%), whereas controls were predominantly from Semey (63%). Employment status also differed significantly (*p* < 0.001), with a higher percentage of controls currently not employed (69%) compared to GDM participants (35%). Conversely, a greater proportion of GDM patients reported being employed (40% vs. 18%). Family history of diabetes did not differ significantly between groups (*p* = 0.062), nor did gravidity (median gravidity = 2.0 for both groups; *p* = 0.47). However, parity showed a significant difference (*p* = 0.009), with a higher proportion of controls being multiparous (89% vs. 78%). Smoking status was comparable across groups (*p* = 0.090), with the majority of both groups being non-smokers.

Table 2 presents the results of the HWE analysis conducted in the control group using the exact test. Genotype distributions for all investigated SNPs were consistent with HWE expectations (exact *p* ≥ 0.05), indicating no evidence of significant deviation from equilibrium and supporting the reliability of genotyping for these variants. The only exception was *IRS1* rs1801278, which significantly deviated from HWE (*p* < 0.0001) and was therefore excluded from further association analyses.

Table 3 presents the associations between selected SNPs and the risk of GDM, based on univariate and multivariate logistic regression analyses adjusted for BMI, education, region, employment status, and parity. In univariate analysis, MTNR1B rs10830963 demonstrated a statistically significant association under the additive model (OR 0.61, 95% CI 0.42–0.89), indicating a potential protective effect of the C allele. However, this association was attenuated and no longer statistically significant after multivariable adjustment (adjusted OR 0.58, 95% CI 0.32–1.03) and correction for multiple testing. In the adjusted analysis, TCF7L2 rs7903146 showed a significant association under the dominant model (adjusted OR 2.29, 95% CI 1.01–5.46); however, this finding did not remain significant following FDR correction. No statistically significant associations were observed for the remaining SNPs under additive, dominant, or recessive genetic models after adjustment for covariates and multiple testing.

Table 4 presents the top-performing gene–gene interaction models identified using 10-fold cross-validation. The best two-locus model included *IGF2BP2* rs4402960 and *CDKAL1* rs7754840 (CVC = 6/10; testing accuracy = 0.558; permutation *p* < 0.001). The most stable interaction was observed for the three-locus model comprising *IGF2BP2* rs4402960, *MTNR1B* rs10830963, and *PPARG* rs1801282 (CVC = 9/10; testing accuracy = 0.576; permutation *p* < 0.001). Although statistically significant, the modest testing accuracy suggests limited predictive capacity.

## 4. Discussion

This study did not identify any SNP that remained significantly associated with GDM after correction for multiple testing. Although *MTNR1B* rs10830963 demonstrated a statistically significant association under the additive model in univariate analysis, this association did not remain significant after adjustment. Similarly, *TCF7L2* rs7903146 showed a nominally significant association under the dominant model in adjusted analysis; however, this finding also did not persist after correction for multiple testing. No statistically robust independent association was observed for other investigated variants.

These findings suggest that common variants in *IGF2BP2*, *CDKAL1*, *MTNR1B, TCF7L2*, *PPARG*, and *GCK* do not exert strong independent effects on GDM susceptibility in this cohort of Kazakh women. The suggestive signal observed for *MTNR1B* rs10830963 is biologically plausible, given the established role of *MTNR1B* in glucose homeostasis and β-cell function [35]. Variants in *MTNR1B* have been associated with fasting glucose levels and type 2 diabetes risk in multiple populations [36,37]. Likewise, *TCF7L2* rs7903146 is one of the most consistently replicated loci for type 2 diabetes [37,38]. The observed effect sizes were modest and confidence intervals were wide, consistent with limited statistical power to detect small genetic effects typical of complex metabolic disorders.

Beyond SNP analyses, MDR analysis suggested potential gene–gene interaction models involving *IGF2BP2*, *CDKAL1*, *MTNR1B*, *PPARG*, and *GCK.* While several interaction models demonstrated statistical significance in permutation testing, their overall testing accuracy was modest (≤0.58), indicating limited predictive capacity. Thus, these interaction findings should be regarded as exploratory rather than confirmatory.

Taken together, the updated results do not support a strong independent genetic determinant of GDM among the investigated candidate variants in this population. Instead, the data are more consistent with a polygenic architecture characterized by modest individual effects and possible weak epistatic interactions. Given the moderate sample size and the typical small effect sizes of common metabolic variants (OR ≈ 1.1–1.3), the study was likely underpowered to detect subtle associations after correction for multiple testing.

Ethnic background and population structure may further influence genetic associations. The Kazakh population has a complex genealogical structure shaped by historical migration patterns and zhuz affiliation. While genealogical differences between cases and controls were observed, multivariable regression models were not adjusted for zhuz affiliation due to substantial multicollinearity between zhuz and region. Our findings suggest that any potential relationship between ancestry-related structure and GDM risk requires further investigation in larger, well-powered cohorts with more balanced regional and genealogical representation.

Our results extend existing evidence. Previous case–control research in China reported no significant association between *IGF2BP2* polymorphisms and GDM, a finding that was consistent with subsequent meta-analytic results showing no association across multiple genetic models [21]. In line with this evidence, a study from Poland involving women with gestational diabetes mellitus and those with normal glucose tolerance found no association between *IGF2BP2* rs4402960 and rs11705701 variants and the risk of GDM [39]. A study conducted in Russia also found no association between the *IGF2BP2* rs4402960 variant and the risk of GDM [40]. Genetic background, including ethnicity, plays an important role in those associations.

A prior study reported marked ethnic differences in genetic susceptibility to gestational diabetes mellitus. Among White women, GDM was associated with rs7901695 variants in *TCF7L2*, rs10830963 variants in *MTNR1B*, and rs780094 variants in *GCKR*, which are also linked to T2DM and fasting glucose regulation in non-pregnant populations [41]. In contrast, among African American women, an elevated risk was observed in individuals homozygous for the *TSPAN8* rs7961581 C allele, whereas carriers of the *JAZF1* rs864745 T allele demonstrated a reduced risk [41]. The protective association observed for the TT genotype compared with the GG and GT genotypes of the *IGF2BP2* rs4402960 polymorphism in our study contrasts with the findings reported in previous studies. These discrepancies may be explained by ethnic-specific linkage disequilibrium patterns, gene–environment interactions, or differences in diagnostic criteria. The lack of significant associations for all investigated SNPs highlights the need for population-specific research and may reflect underlying genetic heterogeneity.

In Kazakhstan, projections indicate a decline in type 2 diabetes prevalence—from 3758 cases in 2020 to 857 by 2030—suggesting possible improvements in public health outcomes [32]. However, GDM continues to impose a significant and enduring economic and social burden, affecting both maternal and offspring health across generations. In this context, leveraging all available tools for early detection, risk stratification, and prevention of GDM is paramount. Although our study did not identify robust independent genetic associations after correction for multiple testing, the observed suggestive signals and exploratory interaction patterns highlight the complexity of GDM susceptibility in this population. Rather than supporting immediate clinical integration of specific genetic markers, our findings underscore the importance of cautious interpretation and the need for larger, well-powered studies before genetic information can be incorporated into screening strategies. By recognizing the genetic diversity and population substructure specific to Kazakhstan, particularly among ethnic Kazakh women, future research may better clarify whether modest polygenic effects or gene–environment interactions contribute meaningfully to GDM risk. The consideration of zhuz-related ancestral clustering as both a potential source of genetic insight and a source of confounding remains essential to advancing genomic research in populations with complex genealogical structures. At present, however, the clinical utility of the investigated SNPs for risk stratification appears limited. Future research should build on these findings by incorporating larger cohorts, longitudinal designs, and multi-omics approaches to further unravel the complex interplay between genetics, ancestry, and metabolic health during pregnancy.

Despite its strengths, this study has several limitations. The sample size was determined by accrual rather than by an a priori power calculation, resulting in modest statistical power and limited ability to detect small effect sizes typical of common genetic variants, thereby increasing the risk of type II error. Regional and genealogical imbalances between cases and controls may have introduced residual confounding, and regression models were not adjusted for zhuz affiliation due to substantial multicollinearity with region, limiting the ability to fully disentangle ancestry-related effects. Although nominal associations were observed in selected models, none remained significant after correction for multiple testing, and the predefined candidate SNP approach may have overlooked population-specific loci. Additionally, MDR interaction findings demonstrated modest predictive accuracy and should be considered exploratory. The absence of statistically significant associations after correction for multiple testing may reflect limited power rather than the absence of a biological effect. Finally, the observational design precludes causal inference, and replication in larger, ancestry-aware cohorts with genome-wide approaches is warranted.

## 5. Conclusions

The findings suggest that common variants in *IGF2BP2*, *CDKAL1*, *MTNR1B*, *TCF7L2*, *PPARG*, and *GCK* do not exert strong individual effects on GDM susceptibility in this cohort of ethnic Kazakh women. Instead, the results are more consistent with a modest polygenic architecture characterized by small effect sizes and possible weak gene–gene interactions. MDR analysis identified statistically significant interaction models; however, their limited predictive accuracy indicates that these findings should be interpreted as exploratory.

Taken together, the present study does not support the immediate clinical utility of the investigated SNPs for GDM risk prediction or screening in the Kazakh population. Nevertheless, the observed suggestive patterns and the unique genealogical structure of this population underscore the importance of larger, ancestry-aware, and methodologically robust studies. Future research incorporating genome-wide approaches, formal adjustment for population structure, and functional validation will be essential to clarify the genetic contribution to GDM and to determine whether polygenic or ancestry-specific effects meaningfully influence disease risk.

## Figures and Tables

**Table 1:** Baseline characteristics of study participants.

Variable	GDM *N* = 217	Control *N* = 148	*p*-Value
Age (years), median [Q1, Q3]	32.0 [27.0, 35.0]	30.0 [27.0, 35.0]	0.59
BMI, median [Q1, Q3]	29.49 [24.52, 34.08]	25.34 [21.77, 31.2]	0.0187
Maternal grandmother genealogical zhuz, *n* (%)			<0.001
Kozha or Tore	4 (1.8%)	3 (2.0%)	
Kishi	115 (53%)	11 (7.4%)	
Orta	54 (25%)	110 (74%)	
Uly	44 (20%)	24 (16%)	
Maternal grandfather genealogical zhuz, *n* (%)			<0.001
Kozha or Tore	4 (1.8%)	3 (2.0%)	
Kishi Zhuz	115 (53%)	11 (7.4%)	
Orta	55 (25%)	111 (75%)	
Uly	43 (20%)	23 (16%)	
Paternal grandmother genealogical zhuz, *n (%)*			<0.001
Kozha or Tore	5 (2.3%)	3 (2.0%)	
Kishi	113 (52%)	12 (8.2%)	
Orta	58 (27%)	109 (74%)	
Uly	41 (19%)	23 (16%)	
Paternal grandfather genealogical zhuz, *n* (%)			<0.001
Kozha or Tore	5 (2.3%)	3 (2.0%)	
Kishi	115 (53%)	12 (8.1%)	
Orta	57 (26%)	110 (74%)	
Uly	40 (18%)	23 (16%)	
Marital status, *n* (%)			0.242
Married	203 (94%)	140 (97%)	
Not married	14 (6.5%)	5 (3.4%)	
Education, *n* (%)			0.038
High school	26 (12%)	8 (5.5%)	
Technical	73 (34%)	42 (29%)	
Bachelor’s and higher	118 (54%)	95 (66%)	
Region, *n* (%)			<0.001
Aktobe	96 (44%)	4 (2.7%)	
Almaty	49 (23%)	36 (24%)	
Almaty Region	59 (27%)	15 (10%)	
Semey	13 (6.0%)	93 (63%)	
Employment, *n* (%)			<0.001
Currently not employed	77 (35%)	100 (69%)	
Employed	87 (40%)	26 (18%)	
Not employed	53 (24%)	19 (13%)	
Family history of diabetes, *n* (%)			0.062
Do not know	1 (0.5%)	1 (0.7%)	
No	171 (79%)	127 (87%)	
Yes	45 (21.1%)	11 (11.7%)	
Gravidity (excl. current), median [Q1, Q3]	2.0 [1.0, 4.0]	2.0 [1.0, 4.0]	0.47
Parity, *n* (%)			0.009
Multiparae	163 (78%)	115 (89%)	
Nulliparae	47 (22%)	14 (11%)	
Smoking status, *n* (%)			0.090
Current smoker	7 (3.2%)	0 (0%)	
No	205 (94%)	141 (97%)	
Stopped during this pregnancy	5 (2.3%)	4 (2.8%)	

Abbreviations: BMI—body mass index; GDM—gestational diabetes mellitus.

**Table 2:** Hardy–Weinberg Equilibrium in control group (exact test).

SNP	*N*	Genotype 1	Obs.	Genotype 2	Obs.	Genotype 3	Obs.	HWE *p*-Value	HWE Status
*IGF2BP2* rs1470579	134	AA	17	AC	66	CC	51	0.58509	In HWE (Exact *p* ≥ 0.05)
*IGF2BP2* rs4402960	122	GG	14	GT	62	TT	46	0.43573	In HWE (Exact *p* ≥ 0.05)
*CDKAL1* rs7754840	122	CC	17	GC	55	GG	50	0.84493	In HWE (Exact *p* ≥ 0.05)
*IRS1* rs1801278	134	CT	6	TC	6	TT	122	<0.0001	Deviates from HWE
*MTNR1B* rs10830963	134	CC	16	GC	63	GG	55	0.85112	In HWE (Exact *p* ≥ 0.05)
*TCF7L2* rs7903146	134	CC	4	TC	37	TT	93	0.76547	In HWE (Exact *p* ≥ 0.05)
*PPARG* rs1801282	134	CC	2	GC	34	GG	98	1.00000	In HWE (Exact *p* ≥ 0.05)
*GCK* rs4607517	133	AA	5	AG	45	GG	83	1.00000	In HWE (Exact *p* ≥ 0.05)

Abbreviations: *CDKAL1*—Cyclin-Dependent Kinase 5 Regulatory Subunit-Associated Protein 1-Like; *GCK*—Glucokinase; HWE—Hardy–Weinberg Equilibrium; *IGF2BP2*—Insulin-Like Growth Factor 2 mRNA-Binding Protein 2; *IRS1*—Insulin Receptor Substrate 1; *MTNR1B*—Melatonin Receptor 1B; *N*—Number of Participants; Obs.—Observed Genotype Count; *PPARG*—Peroxisome Proliferator-Activated Receptor Gamma; SNP—Single-Nucleotide Polymorphism; *TCF7L2*—Transcription Factor 7-Like 2.

**Table 3:** Univariate and multivariate logistic regression of SNPs with gestational diabetes.

Gene	Model	Genotypes	GDM	Control	OR (95% CI)	Adjusted OR (95% CI)	FDR *p*-Value
*IGF2BP2* rs1470579	Additive	CC/CA/AA (per A allele)	23/81/55	16/54/31	0.89 (0.61–1.29)	0.58 (0.32–1.04)	0.7504
*IGF2BP2* rs1470579	Dominant	CA + AA vs. CC			0.84 (0.49–1.42)	0.49 (0.22–1.07)	
*IGF2BP2* rs1470579	Recessive	AA vs. CC + CA			0.9 (0.45–1.82)	0.55 (0.16–1.76)	
*IGF2BP2* rs4402960	Additive	TT/TG/GG (per G allele)	27/82/50	12/49/31	1.15 (0.78–1.69)	0.87 (0.49–1.53)	0.7504
*IGF2BP2* rs4402960	Dominant	TG + GG vs. TT			1.11 (0.64–1.91)	0.66 (0.28–1.48)	
*IGF2BP2* rs4402960	Recessive	GG vs. TT + TG			1.36 (0.67–2.93)	1.27 (0.44–3.81)	
*CDKAL1* rs7754840	Additive	GG/GC/CC (per C allele)	13/63/64	10/41/41	0.94 (0.63–1.41)	0.97 (0.55–1.7)	0.7972
*CDKAL1* rs7754840	Dominant	GC + CC vs. GG			0.95 (0.56–1.62)	0.89 (0.42–1.89)	
*CDKAL1* rs7754840	Recessive	CC vs. GG + GC			0.84 (0.35–2.05)	1.16 (0.34–4.08)	
*MTNR1B* rs10830963	Additive	GG/GC/CC (per C allele)	14/60/85	14/50/37	0.61 (0.42–0.89)	0.58 (0.32–1.03)	0.0759
*MTNR1B* rs10830963	Dominant	GC + CC vs. GG			0.5 (0.3–0.84)	0.45 (0.2–1)	
*MTNR1B* rs10830963	Recessive	CC vs. GG + GC			0.6 (0.27–1.33)	0.59 (0.18–1.89)	
*TCF7L2* rs7903146	Additive	TT/TC/CC (per C allele)	4/61/92	4/29/68	1.28 (0.81–2.05)	1.79 (0.91–3.68)	0.7504
*TCF7L2* rs7903146	Dominant	TC + CC vs. TT			1.46 (0.87–2.47)	2.29 (1.01–5.46)	
*TCF7L2* rs7903146	Recessive	CC vs. TT + TC			0.63 (0.15–2.74)	0.98 (0.13–6.7)	
*PPARG* rs1801282	Additive	GG/GC/CC (per C allele)	3/41/111	1/27/73	1.07 (0.64–1.81)	1.3 (0.59–2.99)	0.7972
*PPARG* rs1801282	Dominant	GC + CC vs. GG			1.03 (0.59–1.82)	1.45 (0.6–3.57)	
*PPARG* rs1801282	Recessive	CC vs. GG + GC			1.97 (0.25–40.21)	0.62 (0.04–16.07)	
*GCK* rs4607517	Additive	GG/GA/AA (per A allele)	1/52/103	2/36/62	0.81 (0.5–1.33)	0.78 (0.37–1.61)	0.7504
*GCK* rs4607517	Dominant	GA + AA vs. GG			0.84 (0.5–1.42)	0.79 (0.35–1.77)	
*GCK* rs4607517	Recessive	AA vs. GG + GA			0.32 (0.01–3.34)	0.46 (0.02–6.31)	

Abbreviations: *CDKAL1*—Cyclin-Dependent Kinase 5 Regulatory Subunit-Associated Protein 1-Like; CI = Confidence Interval; FDR = False Detection Rate; *GCK*—Glucokinase; *IGF2BP2*—Insulin-Like Growth Factor 2 mRNA-Binding Protein 2; GDM—Gestational Diabetes Mellitus; *MTNR1B*—Melatonin Receptor 1B; OR—Odds Ratio; *PPARG*—Peroxisome Proliferator-Activated Receptor Gamma; SNP—Single-Nucleotide Polymorphism; *TCF7L2*—Transcription Factor 7-Like 2. Adjusted for BMI, education, region, employment status, and parity.

**Table 4:** Best MDR gene–gene interaction models (10-fold CV).

Locus No.	Best_Combination	CVC	Testing Accuracy	*p* Value
2	*IGF2BP2* rs4402960: *CDKAL1* rs7754840	6	0.558	0
3	*IGF2BP2* rs4402960: *MTNR1B* rs10830963: *PPARG* rs1801282	9	0.576	0
4	*CDKAL1* rs7754840: *MTNR1B* rs10830963: *PPARG* rs1801282: *GCK* rs4607517	9	0.581	0

## Data Availability

The original contributions presented in this study are included in this article. Further inquiries can be directed to the corresponding authors (B.S., and I.K.).

## Supplementary Materials

The following supporting information can be downloaded at https://www.mdpi.com/article/10.3390/diagnostics16050663/s1: Table S1: Clinical and genetic data of study participants analyzed in the study.

## Abbreviations

The following abbreviations are used in this manuscript:

ALT	Alanine Aminotransferase
AOR	Adjusted Odds Ratio
AST	Aspartate Aminotransferase
BMI	Body Mass Index
CI	Confidence Interval
CVC	Cross-Validation Consistency
DALY	Disability-Adjusted Life Year
DM	Diabetes Mellitus
GDM	Gestational Diabetes Mellitus
HDL	High-Density Lipoprotein
IQR	Interquartile Range
LDL	Low-Density Lipoprotein
MDR	Multifactor Dimensionality Reduction
OGTT	Oral Glucose Tolerance Test
OR	Odds Ratio
PCR	Polymerase Chain Reaction
QALY	Quality-Adjusted Life Year
RBCs	Red Blood Cells
SD	Standard Deviation
SDI	Socio-demographic Index
SNP	Single-Nucleotide Polymorphism
T2DM	Type 2 Diabetes Mellitus
TC	Total Cholesterol
TE buffer	Tris-EDTA Buffer (pH 8.0, used in molecular biology)
TG	Triglycerides
TTG	Tissue Transglutaminase Antibodies
WHO	World Health Organization
